# A cross-sectional and longitudinal evaluation of serum creatinine as a biomarker in spinal muscular atrophy

**DOI:** 10.1186/s13023-024-03515-0

**Published:** 2024-12-25

**Authors:** Xin Zhao, Zhenxiang Gong, Han Luo, Zehui Li, Rong Gao, Kangqin Yang, Wenhua Deng, Sirui Peng, Li Ba, Yang Liu, Min Zhang

**Affiliations:** 1 Department of Neurology, Tongji Hospital, Tongji Medical College, Huazhong University of Science and Technology, Wuhan, 430030 Hubei province China; 2 Department of Neurology, Shanxi Bethune Hospital, Shanxi Academy of Medical Sciences, Tongji Shanxi Hospital, Third Hospital of Shanxi Medical University, Taiyuan, 030032 Shanxi province China

**Keywords:** Spinal muscular atrophy, Creatinine, Biomarkers, Nusinersen

## Abstract

**Objective:**

Spinal muscular atrophy (SMA) is an autosomal recessive neuromuscular disease characterized by proximal muscle weakness and atrophy. The increasing availability of disease-modifying therapies has prompted the development of biomarkers to facilitate clinical assessments. We explored the association between disease severity and serum creatinine (Crn) levels in SMA patients undergoing up to two years of treatment with nusinersen.

**Methods:**

We measured serum Crn levels and assessed function performance using the Hammersmith Functional Motor Scale-Expanded (HFMSE), Medical Research Council Scale (MRC), 6-Minute Walk Test (6MWT), ulnar Compound Muscle Action Potential (CMAP), and forced vital capacity (FVC) in a cohort comprising 28 adolescent and adult patients with SMA. The association between Crn and disease severity was investigated through partial rank correlation analysis and linear mixed models while accounting for age, gender, and BMI. Linear models were employed to predict functional performance.

**Results:**

28 SMA patients and 28 gender- and age-matched healthy controls (HCs) were included, resulting in a dataset of 185 visits. Compared to HCs, SMA patients exhibited significantly lower Crn values ​​(67.4 ± 14 vs. 23.7 ± 14.8 umol/L, *p*<0.0001). After adjusting for age, sex, and BMI, Crn showed positive correlations with the HFMSE (*p*<0.0001, *r* = 0.884), MRC (*p*<0.0001, *r* = 0.827), FVC (*p* = 0.002, *r* = 0.730), and ulnar CMAP (*p*<0.0001, *r* = 0.807). Patients with SMN2 copy number ≥ 4 had nearly twice as high Crn levels as those with SMN2 copy number < 4 (34.1 ± 3.75 vs. 17.2 ± 2.52 umol/L, *p* = 0.00145). Ambulant SMA patients had more than double the Crn levels compared to non-ambulant ones (32 ± 2.33 vs. 12.9 ± 2.38 umol/L, *p*<0.0001). Furthermore, Crn explained that up to 83.5% of the variance in functional performance measured by HFMSE, MRC, and 6MWT was significantly higher than that of traditional biomarkers.

**Conclusions:**

These findings suggest that Crn may be a potential biomarker for assessing disease severity in adolescents and adults with SMA, demonstrating its promise in clinical applications.

**Supplementary Information:**

The online version contains supplementary material available at 10.1186/s13023-024-03515-0.

## Introduction

Spinal muscular atrophy (SMA) is an autosomal recessive neuromuscular disease affecting motor neurons in the spinal cord and brainstem [1]. SMA represents the most prevalent monogenic cause of infant mortality, characterized by symmetrical proximal muscle weakness, muscle atrophy, diminished or absent tendon reflexes, and potential association with scoliosis and progressively restrictive respiratory dysfunction [1, 2]. The incidence of SMA is 5–13 cases per 100,000 live births [3]. Biallelic deletion or mutations of the *survival motor neuron 1* (*SMN1*) gene account for approximately 95% of SMA cases; other genes contribute to the remaining 5% [1, 2]. Pathogenic SMN1 mutations have a carrier frequency ranging from 1:100 to 1:45. The modified SMN2 gene shares over 99% identical sequences with SMN1. It can produce partially functional full-length SMN protein, which is implicated in disease phenotypes [4]. The clinical severity of SMA often exhibits an inverse correlation with the copy number of the SMN2 gene. Based on the age of onset and the attainment of movement milestones, SMA can be divided into types 0 to 4 [5].

Nusinersen, the first approved disease-modifying therapy for SMA, has received marketing approval in numerous countries worldwide [6, 7]. It exerts its effects by modifying the splicing of the SMN2 gene to enhance the synthesis of normal full-length SMN protein [7]. Given the heterogeneity in response to Nusinersen treatment, biomarkers are urgently needed to detect and quantify disease progression accurately. The current methods used to evaluate efficacy primarily rely on motor function scores, susceptible to evaluator subjectivity and ceiling and floor effects associated with the scoring scale [7–9]. Existing SMA biomarkers encompass various dimensions, including genetic indicators (e.g., SMN2 copy number), molecular markers (e.g., SMN protein and neurofilament protein), electrophysiological indices such as compound muscle action potential (CMAP), motor unit number estimation (MUNE), motor unit index (MUNIX), and electrical impedance myography (EIM)), as well as imaging markers like MRI and muscle ultrasound [10–14]. However, these biomarkers need further refinement regarding accuracy in reflecting disease progression and increased sensitivity for effectively capturing therapeutic response. Additionally, acquiring and detecting these biomarkers presents significant challenges. Hence, it is imperative to identify biomarkers demonstrating high sensitivity, reliability, and accessibility for monitoring treatment efficacy and disease progression.

Creatinine (Crn) is a byproduct of muscle metabolism and is directly associated with muscle mass, activity, and metabolic rate [15]. Therefore, monitoring Crn levels and their fluctuations can be a fundamental basis for disease diagnosis, treatment, and prognosis assessment. A recent study investigated serum Crn levels in SMA patients and identified a correlation between disease progression and denervation extent [16]. However, this study did not involve individuals with adolescent or adult-onset SMA or disease-modifying treatments. Although another cross-sectional study exploring the potential of serum Crn as a biomarker for disease severity in adults with SMA types 2 and 3 [17], establishing the scientific validity of serum Crn through cross-sectional studies remains challenging due to the progressive nature of SMA. Therefore, further validation is required to determine whether serum Crn levels can be a reliable biomarker for assessing disease severity in adolescents and adults with SMA. Moreover, additional studies are warranted to explore the potential of serum Crn levels in predicting drug efficacy. This study aimed to evaluate the utility of Crn as a biomarker while investigating its dynamic changes during nusinersen treatment.

## Methods

### Study approval and participants

This prospective, longitudinal, monocentric, observational study enrolled a total of 28 patients (aged 16–51 years old) diagnosed with 5q SMA at the Department of Neurology, Tongji Hospital, Tongji Medical College of Huazhong University of Science and Technology from February 2022 to February 2024. All patients received regular intrathecal nusinersen treatment for up to 2 years following standard procedures. The copy numbers SMN1 and SMN2 were determined using multiplex ligation-dependent probe amplification (MLPA). A comprehensive medical evaluation was conducted for all patients, including a collection of patient demographics and clinical data such as age, sex, baseline weight, height, BMI, clinical subtype based on the Clinical Practice Guideline for Adolescent & Adult Patients with Spinal Muscular Atrophy criteria and SMN2 copy number. Due to the small sample size in patients with 2 SMN2 copies (*n* = 3), they were combined with those having 3 SMN2 copies (SMN2 copy number <4) purposes. Forced vital capacity (FVC) and ulnar CMAP amplitude were assessed at the baseline. Motor function scores were evaluated by two experienced doctors using the Hammersmith Functional Motor Scale-Expanded (HFMSE), Medical Research Council Scale (MRC), and 6-Minute Walk Test (6MWT) before each intrathecal nusinersen injection. Higher scores indicate better motor function. Serum Crn levels were measured enzymatically at the Laboratory Department of Tongji Hospital. This study obtained approval from the Ethics Committee of Tongji Hospital, Tongji Medical College, Huazhong University of Science and Technology, adhering to the principles outlined in the Declaration of Helsinki (2022-S184) [18]. Informed consent was obtained from either the patients or their legal representatives. Furthermore, a group of 28 sex- and age-matched healthy controls (HC) were included in this study, and their serum Crn values were recorded.

### Statistical analysis

The quantitative data, including motor function scores, Crn, FVC, and CMAPs, were presented as standard deviation (SD). Wilcoxon test was used to compare Crns between SMA patients and healthy controls, while Mann-Whitney U test was employed to compare Crn between patients with SMN2 copy number <4 and patients with SMN2 copy number ≥ 4. Partial rank correlation was conducted to correct for age, gender, and BMI and analyze the correlation between Crns and HFMSE, MRC, 6MWT, FVC, and CMAP. A linear mixed-effects model was utilized to establish the relationship between Crns and disease severity by controlling for age, sex, and BMI. To compare Crn levels among participants with SMN2 copies <4 and ≥ 4 in terms of ambulatory status, the following model was applied: Crns = A + B1(i) × SMN2 + B2 × patient age + B3 × patient sex + B4 × BMI + mixed term + error term, where SMN2 copies ≥ 4 was used as the reference group. The prediction of motor function was calculated using linear models incorporating various combinations of variables. To compare Crn levels between ambulant and non-ambulant patients, the model was formulated as follows: Crns = A + B(i) × (non-ambulant patient / ambulant patient) + B2 × patient age + B3 × patient sex + B4 × BMI + mixed term + error term, whit the ambulant patient group serving as the reference. The prediction of motor function was calculated using linear models with various combinations of variables (Crn, FVC, and SMN2 copy) and BMI. The predictive performance was assessed based on R2 vålues obtained through bootstrap resampling. Statistical analysis was performed using IBM SPSS Statistics 24 and Rstudio; figure generation utilized GraphPad Prism 10.

## Results

This study included 28 SMA patients with 185 visits and 28 gender- and age-matched healthy controls. Two SMA patients, aged 15 and 16, are adolescents, and the remaining 26 are adults aged 18 to 50. Of the total 28 patients, 13 cannot walk, with five being unable to sit independently and eight being unable to sit alone. Fifteen patients are capable of walking independently. Eight patients are classified as type 2, 17 as type 3, and three as type 4. Detailed characteristics of patients and controls are shown in Table 1.

### Crn is significantly decreased in patients with SMA

**Fig. 1:**
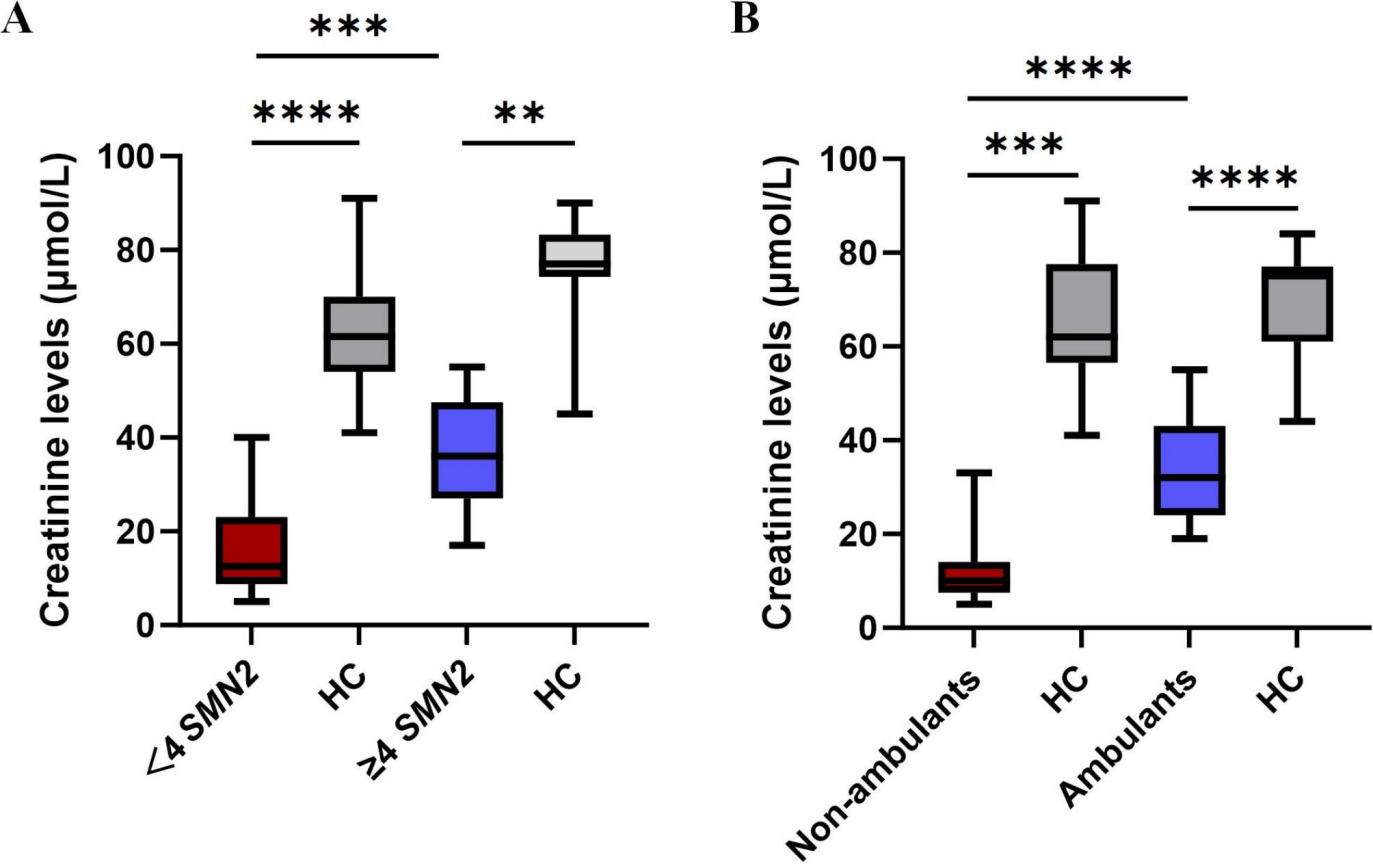
Comparison of Crn values ​​between SMA patients and healthy controls. (**A**) The Crn values ​​of patients with SMN2 copies <4 were significantly lower than those with SMN2 copies ≥ 4. (**B**) The Crn values ​​of non-ambulant patients were significantly lower than those of ambulant ones. **: *p*<0.01;***: *p*<0.001; ***: *p*<0.0001; HC: Healthy controls

Compared to healthy controls, SMA patients had significantly lower Crn values ​​(67.4 ± 14 vs. 23.7 ± 14.8 umol/L, *p*<0.0001) (Table 1). Based on the copy number of SMN2 gene, SMA patients were categorized into two groups: those with less than four copies and those with four or more copies of the SMN2 gene. Comparison of Crn values between ​​the two groups revealed that patients with SMN2 gene copies <4 had significantly lower Crn levels (Median: 12.5 umol/L, Q1-Q3: 8.75-23 umol/L, *n* = 18) compared to those with SMN2 gene copies ≥ 4 (Median: 36 umol/L, Q1-Q3: 27-47.5 umol/L, *n* = 10) (*p* = 0.0003) (Fig. 1A). Moreover, the Crn values ​​of both groups of SMA patients were significantly lower than those of gender- and age-matched healthy controls. Then, patients are categorized into two groups based on their motor function status: ambulant ones who can walk independently and non-ambulant ones who cannot walk independently (including individuals who can sit alone and those who cannot). Comparing the Crn values ​​between the two groups revealed that the Crn values ​​of non-ambulant patients (Median: 10 umol/L, Q1-Q3: 7.5–14 umol/L, *n* = 13) were significantly lower than those of ambulant ones (Median: 32 umol/L, Q1-Q3: 24–43 umol/L, *n* = 15) (*p*<0.0001) (Fig. 1B). Additionally, both groups of SMA patients exhibited significantly lower Crn values than healthy controls matched for gender and age.

**Table 1:** Demographic information and baseline data of patients with SMA

	Patients with SMA (*n* = 28)
Age, y, mean (SD)	27.8 (10)
Age of onset, y, mean (SD)	7.81 (9.91)
Course of the disease, y, mean (SD)	19.97 (10.04)
Sex, F/M	11/17
SMA type (2/3/4), n	8/17/3
SMN2 copies (2/3/≥4), n (%)	3/15/10
Motor performance (non-sitter/sitter/ambulant), n	5/8/15
Baseline HFMSE, mean (SD)	31.7 (23.9)
FVC, % (SD)	79.7 (33.9)
Creatinine, umol/L (SD)	23.7 (14.8)

### Crn correlates with the disease severity of SMA

**Fig. 2:**
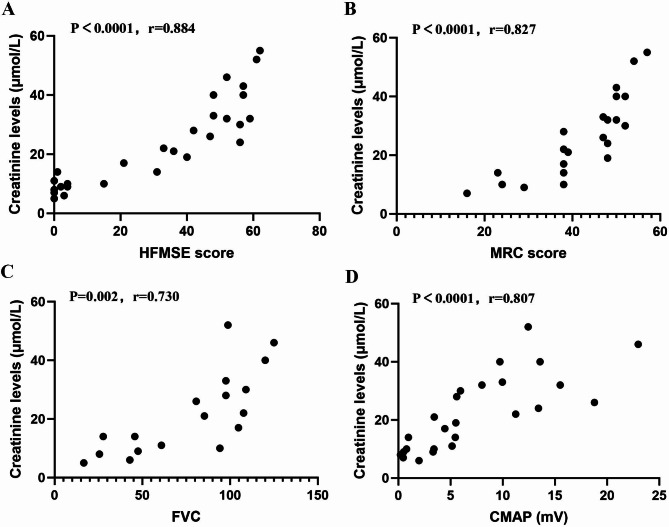
Partial correlation of Crn levels to disease severity among adolescents and adults SMA corrected for age,** sex**,** and BMI**. Crn has a positive correlation with HFMSE (**A**), MRC (**B**), FVC (**C**), and CMAP (**D**) after correction for age, sex, and BMI. HFMSE, Hammersmith Functional Motor Scale Expanded; MRC, Medical Research Council Scale; FVC, forced vital capacity; CMAP, Compound Muscle Action Potential

We employed partial correlation analysis to examine the relationship between Crn and disease severity indicators, including HFMSE, MRC, 6MWT, and FVC. We found that Crn has a positive correlation with HFMSE (*p*<0.0001, *r* = 0.884) and MRC scores (*p*<0.0001, *r* = 0.827), even after adjusting for age, sex, and BMI (Fig. 2A and B). This suggests that as Crn levels increase, there is an improvement in motor function scores and muscle strength among patients. Although the correlation between Crn and 6MWT (*p* = 0.069) was not statistically significant, we observed a trend of increasing Crn levels with higher 6MWT values. Furthermore, our analysis demonstrated a positive relationship between Crn concentration and FVC (*p* = 0.002, *r* = 0.730) after controlling for age, sex, and BMI (Fig. 2C). These results confirm the close connection between Crn levels and respiratory function. Finally, we analyzed the association between Crn and denervation by examining its relationship with ulner CMAP., We found a positive correlation between Crn and ulnar nerve CMAP (*p*<0.0001, *r* = 0.807) after adjusting for age, sex, and BMI (Fig. 2D). This indicates that increased Crn corresponds to an increase in the patient’s ulnar nerve CMAP amplitude.

**Fig. 3:**
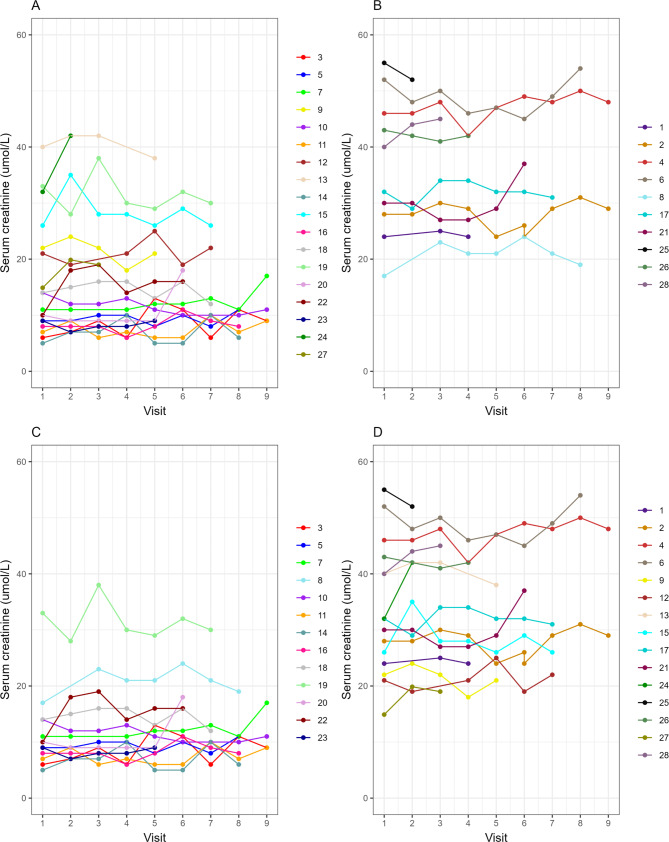
Comparison of creatinine using linear mixed model. Creatinine in patients with SMN2 copy numbers <4 (**A**) is significantly lower than that in patients with SMN2 copy numbers ≥ 4 (**B**). Creatinine in non-ambulant patients (**C**) is substantially lower than in ambulant patients (**D**). Each line represents data from different patients at various follow-up points

Next, we used longitudinally measured Crn values​​ over a follow-up period of up to 2 years to explore the potential association between changing trends in Crn values and disease progression. Initially, we constructed a linear mixed-effects model by comparing the Crn values among patients with SMN2 copy number <4 and those with SMN2 copy number ≥ 4. After adjusting for sex, age, and BMI, our analysis revealed that patients with SMN2 copy number ≥ 4 exhibited significantly higher Crn levels (34.1 ± 3.75 umol/L) compared to patients with SMN2 copy number < 4 (17.2 ± 2.52 umol/L; *p* = 0.00145) (Fig. 3A and B). We further conducted a similar comparison between ambulant and non-ambulant patients. Following adjustment for sex, age, and BMI, we found that ambulant patients had more than twice the Crn level (32 ± 2.33 umol/L; *p*<0.0001) as non-ambulant patients (12.9 ± 2.38 umol/L) (Fig. 3C and D).

To elucidate the impact of nusinersen treatment on serum Crn levels and motor function in patients, we conducted a comprehensive analysis of Crn values at 6 months, 14 months, and 22 months post-treatment (Supplementary Figure S1), as well as changes in the HFMSE scores (Supplementary Figure S2). Our findings revealed that after 6 months of nusinersen treatment, stable or elevated Crn levels were observed in 50% (11/22) of patients; after 14 months, this percentage increased to 68.8% (11/16); and after 22 months, it further rose to 83.3% (5/6) (Supplementary Figure S1). Similarly, the HFMSE scores remained stable or increased in 93.3% (14/15), 100% (6/6), and 85.7% (6/7) of patients at 6 months, 14 months, and 22 months, respectively (Supplementary Figure S2). These results suggest that nusinersen can preserve or enhance motor function in adolescent and adult patients with SMA. Moreover, they reinforce the notion that serum Crn can serve as a reliable biomarker for disease severity in SMA patients.

### Prediction of motor function using Crn, conventional biomarkers, and BMI

**Fig. 4:**
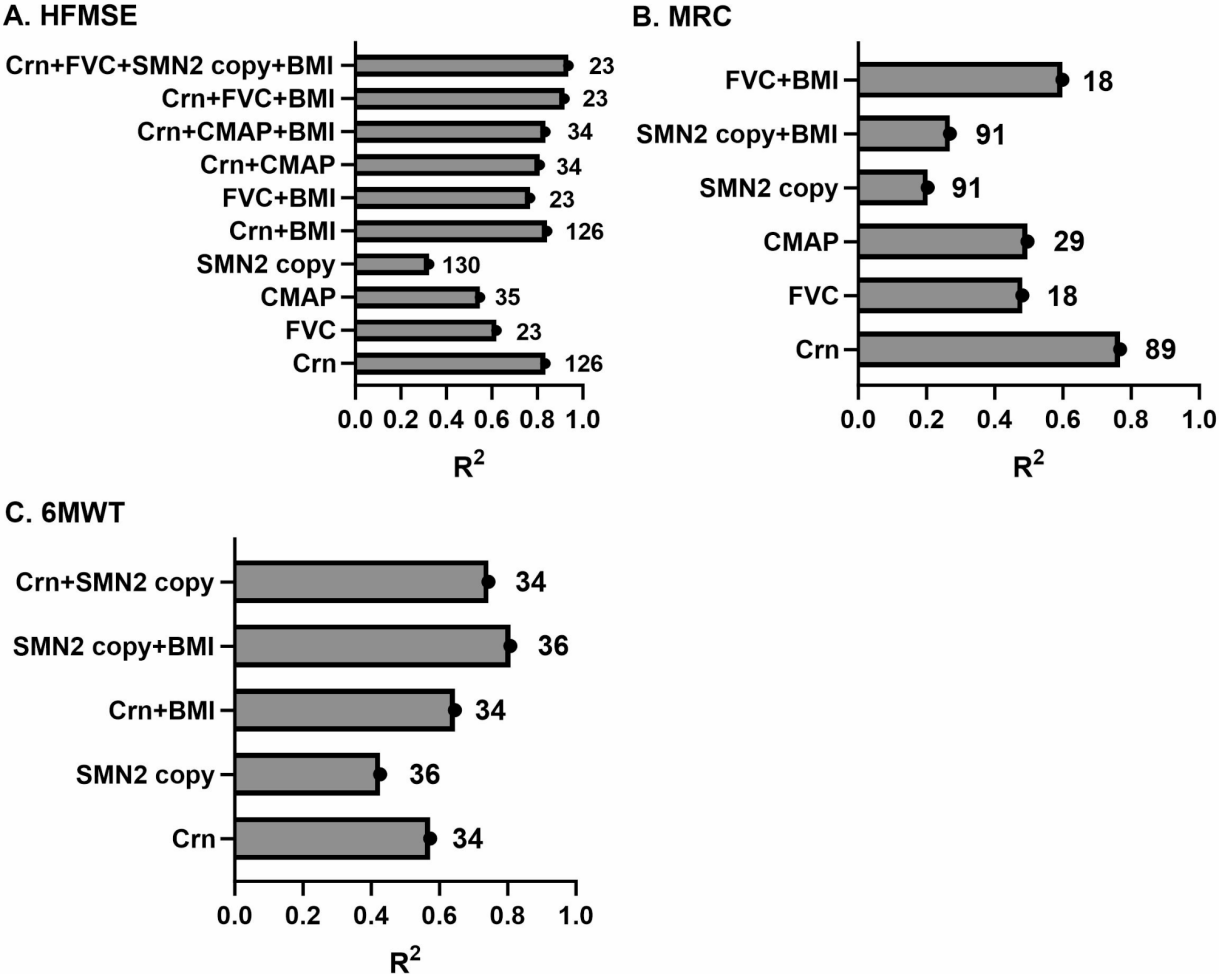
Prediction of motor function scores using Crn, **FVC**,** CMAP**,** SMN2 copy**,** and BMI**. The explained variance of motor function scores, as assessed by HFMSE (**A**), MRC (**B**), and 6MWT (**C**), using each predictor separately, was highest using Crn. The x-axis represents the explained variance, with values closer to 1 indicating a higher degree of variance explained. Numbers behind the bars indicate the quantity of available data points for each prediction

We investigated the predictive capacity of Crn, conventional biomarkers (FVC, CMAP, and SMN2 copy), and BMI on the current motor function assessed by HFMSE, MRC, and 6MWT. When considered individually, Crn exhibited the highest explanatory variance as a predictor, reaching up to 83.5%, significantly superior to other biomarkers (FVC, CMAP, and SMN2 copy) (Fig. 4). Crn and SMN2 copies demonstrated predictive potential for all motor functions, including HFMSE, MRC, and 6MWT. However, FVC and CMAP were inadequate in predicting 6 MW in ambulant SMA patients. By combining Crn with FVC, SMN2 copy, and BMI, the explained variance of HFMSE could be increased to around 93% (Fig. 4A). In terms of predicting 6MWT, the combination of Crn and BMI enhanced the explained variance to around 64%, the combination of Crn and SMN2 copy to around 74%, and the combination of SMN2 copy and BMI to around 80% (Fig. 4C). To investigate the comparative efficacy of Crn, HFMSE, MRC, and 6MWT in evaluating motor function, we employed linear models to assess the correlation between Crn levels and these motor function measures. Our findings indicate that Crn exhibits similar or superior explained variance compared to the scales (Supplementary Figure S3).

## Discussion

In this study, our objective was to comprehensively evaluate the predictive value of serum Crn levels in assessing disease severity among adolescent and adult patients with SMA undergoing nusinersen treatment. Our findings indicate a significantly lower Crn level in adolescent and adult SMA patients than in healthy controls. We observed a strong association between Crn levels and disease severity at baseline and during a two-year follow-up period. Crn accounted for up to 83.55% of the explained variation in motor function. These results highlight the potential utility of Crn as a monitoring biomarker for adolescents and adults with SMA.

The level of Crn is closely associated with muscle mass and metabolic activity [19]. The decreased muscle mass leads to a reduction in Crn production [19]. Therefore, in clinical practice, Crn levels are often used as an indicator to assess muscle status and metabolic function. In this study, all patients exhibited lower Crn levels than the healthy control group due to muscle atrophy. SMN protein deficiency, disrupted fatty acid metabolism, muscle wasting, mitochondrial dysfunction, and compromised nutritional status may decrease Crn levels among SMA patients [20]. By comparing Crn values among patients with different SMN2 copy numbers and varying motor function statuses, we found that patients with SMN2 copy number ≥ 4 showed significantly higher Crn values than those with fewer than four copies. Patients who could walk displayed higher Crn values ​​compared to non-ambulatory patients. In addition, there was a positive correlation between Crn and HFMSE, MRC, FVC, and CMAP. These results suggest that the Crn value is closely related to disease severity and may be a potential biomarker for assessing disease severity and pharmacodynamic efficacy in adolescent and adult SMA patients. Given the progressive nature of SMA, where age is often associated with functional decline, it can be challenging to establish a relationship between biomarkers and functional performance through cross-sectional studies alone. In our study, lower Crn levels were associated with worse motor performance after adjusting for age effects on functional performance. Furthermore, using linear mixed models for longitudinal follow-up analysis revealed an association between changes in Crn levels over time and changes in motor performance, further supporting the relationship between Crn and motor performance. Concurrently, we documented the alterations in serum Crn levels and HFMSE scores of patients over the course of 6, 14, and 24 months of nusinersen therapy. SMA is widely recognized as a relentlessly progressive neurodegenerative disorder. Although some infants and young children may achieve new motor developmental milestones in the early stages of SMA, the overall patient condition tends to deteriorate over time. In our study, most patients exhibited either stable or increased HFMSE scores and Crn levels after treatment, indicating a favorable therapeutic response in these SMA patients. Considering the positive correlation between Crn levels and disease severity, fluctuations in Crn concentrations could serve as a valuable tool for monitoring the natural progression of the disease and evaluating the therapeutic effectiveness of disease-modifying treatments.

One study conducted by Swoboda et al. in 2020, which examined a large cohort, revealed significant differences in serum Crn levels among individuals with SMA who were categorized as walkers, sitters, and non-sitters. The study also found a positive association between Crn levels and maximum CMAP and MUNE, suggesting that Crn may be a potential biomarker for SMA progression [16]. Our findings align with this previous research; however, it is essential to note that the cases included in our study differed from those of the prior investigation. Furthermore, it should be acknowledged that Crn can be influenced by gender, age, BMI, ethnicity, diet, etc [21]. In contrast, the study reported by Swoboda et al. encompassed multiple racial groups, including whites, Asians, blacks, Pacific Islanders, and other unreported patients [16]; our study solely consisted of Han Chinese individuals; thus, race was not considered a confounding factor. Furthermore, the study conducted by Swoboda et al. encompassed a population consisting of infants and children, categorized into types 1, 2, and 3 [16]. In contrast, our present study excluded infants and children, classifying patients into types 2, 3, and 4. Notably, the patients examined in Swoboda et al. ’s research followed a natural disease progression without receiving any disease-modifying treatments [16]. Conversely, the patients in our current study received regular nusinersen treatment, highlighting the predictive role of Crn in disease severity and its impact on treatment efficacy. In 2021, Freigang et al. identified a moderate correlation between serum Crn levels and SMA severity among adult patients classified as types 2 and 3 [17]. However, their investigation solely relied on cross-sectional analyses to assess the relationship between serum Crn levels and baseline disease severity, thus overlooking the value of longitudinal studies. Our research addresses this limitation by incorporating longitudinal data that further strengthens the significance of serum Crn as a reliable biomarker for assessing SMA disease severity.

When considering a single predictor, Crn demonstrated superior accuracy in predicting motor function compared to other markers such as SMN2 copy number, FVC, and CMAP. Using Crn alone accounted for up to 83.55% of the explained variance in motor function. However, employing multiple predictors improved the prediction accuracy of motor function compared to using Crn individually, although this approach may lead to increased time and cost consumption. Despite the limited sample size, our models exhibited high predictive accuracy, indicating that Crn holds promise as a potential biomarker for SMA. This study represents an initial endeavor in utilizing Crn, SMN2 copy number, FVC, CMAP, and BMI to construct a predictive model for motor function in SMA patients. However, further investigations with larger sample sizes are imperative to validate and confirm the accuracy and reliability of our model.

This study has several limitations that necessitate attention. Firstly, the limited number of CMAP and FVC observations may compromise our prediction model’s accuracy for motor function in SMA patients. Therefore, expanding the sample size in future studies is imperative to ensure a more comprehensive analysis. Secondly, we could not control for dietary/supplement intake, which has the potential to influence Crn levels and requires thorough examination in subsequent research. Furthermore, due to the small number of patients with SMN2 copy number 2 included in this study, we had to categorize patients into two groups based on SMN2 copy numbers: less than four and greater than or equal to 4. Similarly, patients who could not sit independently were grouped with those who could sit independently for analysis. The presence of missing data diminishes the adequate sample size, compromising the statistical power and introducing potential bias that may impact the reliability of the results. Broadening the sample size for further analysis and validation in future studies is crucial to enhancing reliability and validity.

## Conclusions

In conclusion, Crn emerges as a promising biomarker for SMA due to its strong association with disease severity and remarkable accuracy in predicting motor function when used alone. However, further investigation involving an extended follow-up period and a larger sample size is imperative to elucidate the longitudinal relationship between Crn and disease progression and treatment efficacy in SMA.

## Electronic supplementary material

Below is the link to the electronic supplementary material.

**Supplementary Material 1**: **Additional information**: Supplementary Figures S1, S2, and S3 and Supplementary Table provides the specific values of each patient’s assessment and serum creatinine levels for each visit are publicly available in the ScienceDB repository: https://www.scidb.cn/en/s/7JBZ7r

## Data Availability

All data supporting the findings of this study are available within the paper and its Supplementary Information.

## Abbreviations

SMA: Spinal muscular atrophy
Crn: Creatinine
HFMSE: Hammersmith functional motor scale-expanded
MRC: Medical research council scale
6MWT: 6-Minute Walk Test
CMAP: Compound muscle action potential
FVC: Forced vital capacity
HC: Healthy control
SMN1: Survival motor neuron 1
MUNE: Motor unit number estimation
MUNIX: Motor unit index
EIM: Electrical impedance myography
MLPA: Multiplex ligation-dependent probe amplification
SD: Standard deviation
