# Supplementary material for: A cross-sectional and longitudinal evaluation of serum creatinine as a biomarker in spinal muscular atrophy
Source: Orphanet J Rare Dis. 2024 Dec 25;19:489. doi: 10.1186/s13023-024-03515-0 (PMC11670464; doi:10.1186/s13023-024-03515-0)
Supplement: Supplementary file 1 — Supplementary Material 1: Additional information: Supplementary Figures S1, S2, and S3 and Supplementary Table provides the specific values of each patient’s assessment and serum creatinine levels for each visit are publicly available in the ScienceDB repository: https://www.scidb.cn/en/s/7JBZ7r [file 13023_2024_3515_MOESM1_ESM.docx]

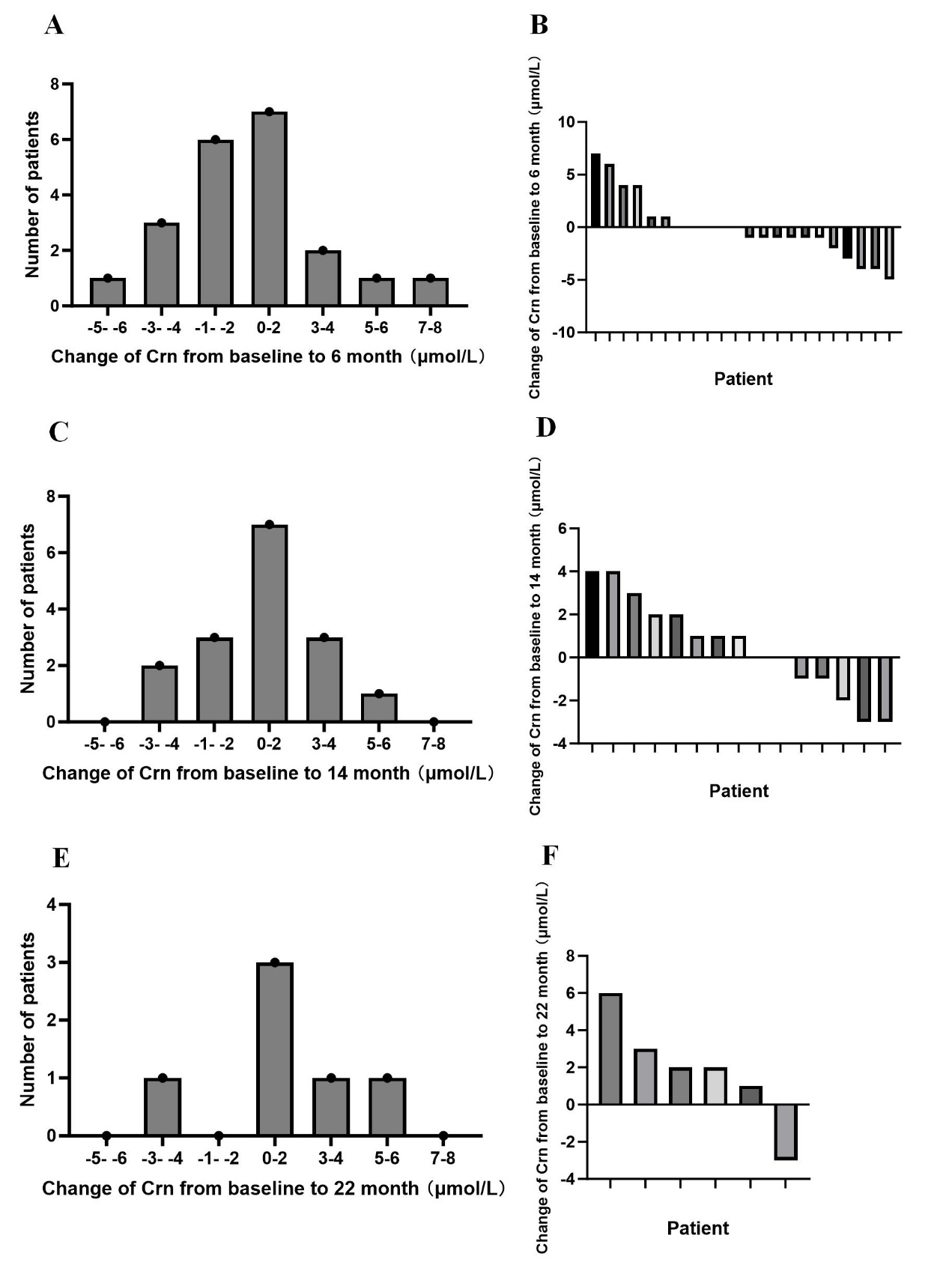


Supplementary Figure S1. Changes in Creatinine level from baseline to 6 (A, B), 14 (C, D), and 22 (E, F) months.

The left panels depict the distribution of changes in Crn levels at 6 (A), 14 (C), and 22 (E) months, with each bar representing the number of patients showing improvement or deterioration. The right panels illustrate individual patient-specific changes in Crn levels from baseline to 6 (B), 14 (D), and 22 (F) months, with each bar representing a single patient.
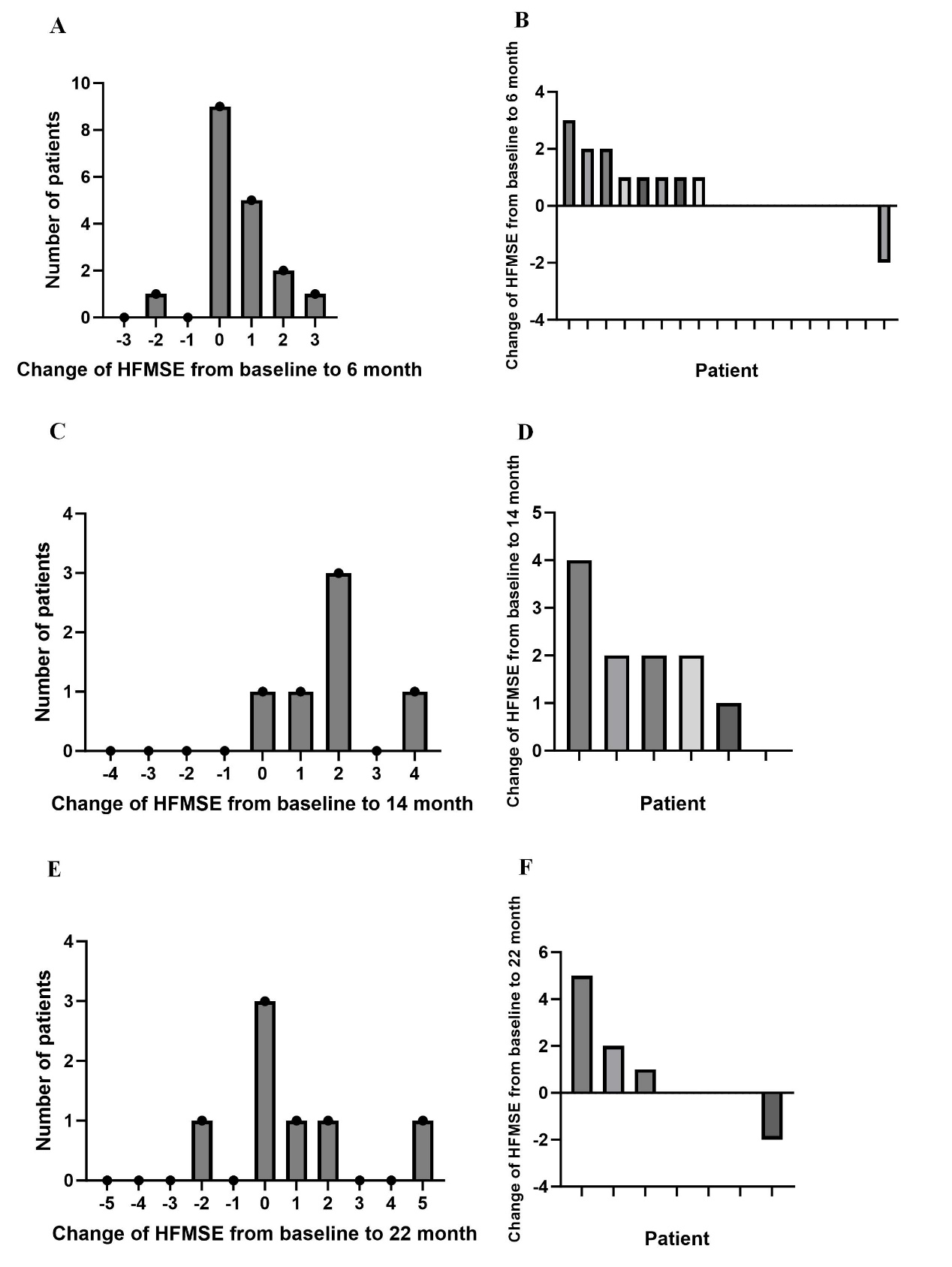


Supplementary Figure S2. Changes in HFMSE scores from baseline to 6 (A, B), 14 (C, D), and 22 (E, F) months.

The left panels depict the distribution of changes in HFMSE scores at 6 (A), 14 (C), and 22 (E) months, with each bar representing the number of patients showing improvement or deterioration. The right panels illustrate individual patient-specific changes in HFMSE scores from baseline to 6 (B), 14 (D), and 22 (F) months, with each bar representing a single patient.


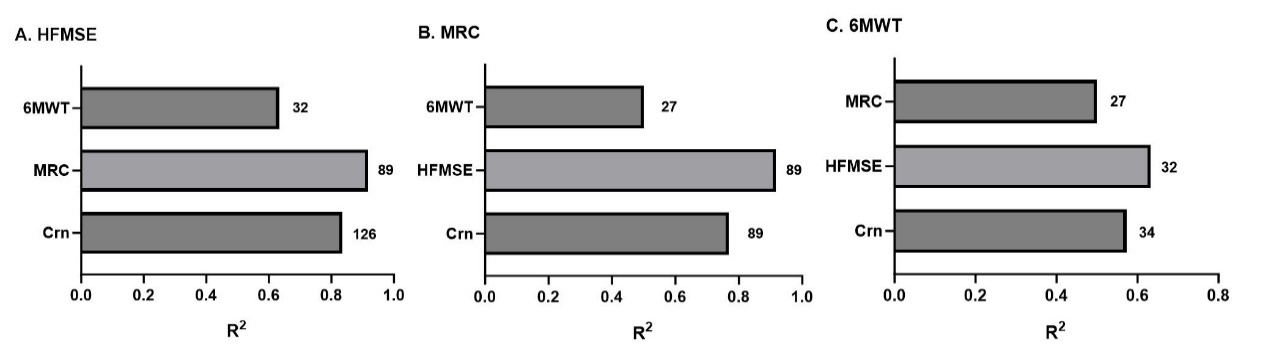


Supplementary Figure S3. Comparison of explained variance between Crn and HFMSE, MRC, and 6MWT.

The explained variance achieved using Crn was comparable to or superior to the scales. The x-axis represents the degree of explained variance, where values closer to 1 indicate a higher variance level. The numbers behind the bars represent the quantity of available data points for each prediction.
